# ETV4 and ETV5 orchestrate FGF-mediated lineage specification and epiblast maturation during early mouse development

**DOI:** 10.1242/dev.204278

**Published:** 2025-03-24

**Authors:** Claire S. Simon, Woonyung Hur, Vidur Garg, Ying-Yi Kuo, Kathy K. Niakan, Anna-Katerina Hadjantonakis

**Affiliations:** ^1^ Developmental Biology Program, Sloan Kettering Institute, Memorial Sloan Kettering Cancer Center, New York, NY 10065, USA; ^2^ Human Embryo and Stem Cell Laboratory, The Francis Crick Institute, 1 Midland Road, London NW1 1AT, UK; ^3^ The Centre for Trophoblast Research, Department of Physiology, Development and Neuroscience, University of Cambridge, Cambridge CB2 3EG, UK; ^4^ Center for Studies in Physics and Biology, the Rockefeller University, New York, NY 10065, USA; ^5^ Wellcome Trust – Medical Research Council Stem Cell Institute, University of Cambridge, Jeffrey Cheah Biomedical Centre, Puddicombe Way, Cambridge CB2 0AW, UK; ^6^ Epigenetics Programme, Babraham Institute, Cambridge CB22 3AT, UK

**Keywords:** ETV, FGF, Primitive endoderm, Epiblast, Pluripotency

## Abstract

Cell fate decisions in early mammalian embryos are tightly regulated processes crucial for proper development. While FGF signalling plays key roles in early embryo patterning, its downstream effectors remain poorly understood. Our study demonstrates that the transcription factors *Etv4* and *Etv5* are crucial mediators of FGF signalling in cell lineage specification and maturation in mouse embryos. We show that loss of *Etv5* compromises primitive endoderm formation at pre-implantation stages. Furthermore, *Etv4* and *Etv5* (*Etv4/5*) deficiency delays naïve pluripotency exit and epiblast maturation, leading to elevated NANOG and reduced OTX2 expression within the blastocyst epiblast. As a consequence of delayed pluripotency progression, *Etv4*/*Etv5-*deficient embryos exhibit anterior visceral endoderm migration defects post-implantation, a process essential for coordinated embryonic patterning and gastrulation initiation. Our results demonstrate the successive roles of these FGF signalling effectors in early lineage specification and embryonic body plan establishment, providing new insights into the molecular control of mammalian development.

## Teaser

**Summary:** FGF signalling effectors ETV4 and ETV5 regulate lineage specification and embryo patterning in mice, affecting primitive endoderm formation and pluripotency exit.

## INTRODUCTION

FGF signalling is essential for mouse pre-implantation development where it acts in multiple successive steps. At the blastocyst stage, within the inner cell mass FGF4 drives the specification of primitive endoderm progenitors, an extra-embryonic endoderm lineage that later forms the yolk sac. Culture in exogenous FGF ligands directs all inner cell mass progenitors to differentiate into primitive endoderm (Nichols et al., 2009; Yamanaka et al., 2010). Conversely, inactivation of FGF signalling through genetic or biochemical means generates embryos with an inner cell mass containing all epiblast cells (Chazaud et al., 2006; Kang et al., 2017, 2013; Krawchuk et al., 2013; Molotkov et al., 2017; Nichols et al., 2009; Yamanaka et al., 2010). However, these epiblast cells have sustained elevated NANOG levels, indicating an inability to exit naïve pluripotency (Kang et al., 2017, 2013; Molotkov et al., 2017; Nichols et al., 2009). FGF signalling is well known to regulate naïve pluripotency exit and later germ-layer differentiation *in vivo* (Lanner and Rossant, 2010; Nichols et al., 2009), and in mouse embryonic stem cells (Kunath et al., 2007; Ying et al., 2008). However, the direct targets of FGF/ERK and the molecular mechanism underlying how FGF/ERK signalling executes cell fate decisions are poorly understood.

*Etv4* and *Etv5*, are ETS transcription factors and downstream transcriptional activators of the FGF signalling pathway during embryonic development, with cooperative roles in the morphogenesis of the lung, limb bud and kidney (Herriges et al., 2015; Zhang et al., 2009). ETS transcription factor expression is induced by RTK/MAPK signalling, and post-translationally activated by MAPK phosphorylation (Charlot et al., 2010; Janknecht et al., 1996; O'Hagan et al., 1996). *Etv4* homozygous mutant males are viable, but sterile (Laing et al., 2000), or have neuronal defects (Livet et al., 2002). Whereas, *Etv5* homozygous mutants die soon after birth (Zhang et al., 2009) or have mid-gestation (E8.5) lethality (Lu et al., 2009), the causes of which have not been established.

Single-cell transcriptomics of mouse (Ohnishi et al., 2014) and human (Blakeley et al., 2015) blastocysts identified expression of *Etv4* (epiblast and primitive endoderm) and *Etv5* (inner cell mass progenitors and epiblast), which are downregulated in *Fgfr1* mutant mouse blastocysts (Kang et al., 2017), suggesting these ETS variant (ETV) proteins may govern the transcriptional output downstream of FGF signalling in the inner cell mass. However, despite being implicated in regulation of the pluripotent state *in vitro* (Akagi et al., 2015; Kalkan et al., 2019; Zhang et al., 2018), the role of these transcription factors during epiblast development *in vivo* has not been assessed.

Here, we investigated the role of *Etv4* and *Etv5* (*Etv4/5*) as potential downstream effectors of FGF signalling in the establishment and maturation of the epiblast lineage. Analysing compound mutant mouse embryos, we find that loss of *Etv5* compromises the formation of the primitive endoderm at pre-implantation stages. At peri-implantation, the loss of *Etv4/5* causes a delay in the progression of pluripotency and epiblast maturation, leading to developmental delay and AVE migration defects at early post-implantation stages. Together, our work sheds light on the successive roles of FGF signalling effectors in mediating inner cell mass fate decisions, and later epiblast maturation required for establishing the embryonic body plan at gastrulation.

## RESULTS

### *Etv4* and *Etv5* expression during mouse embryonic development

**Fig. 1.**
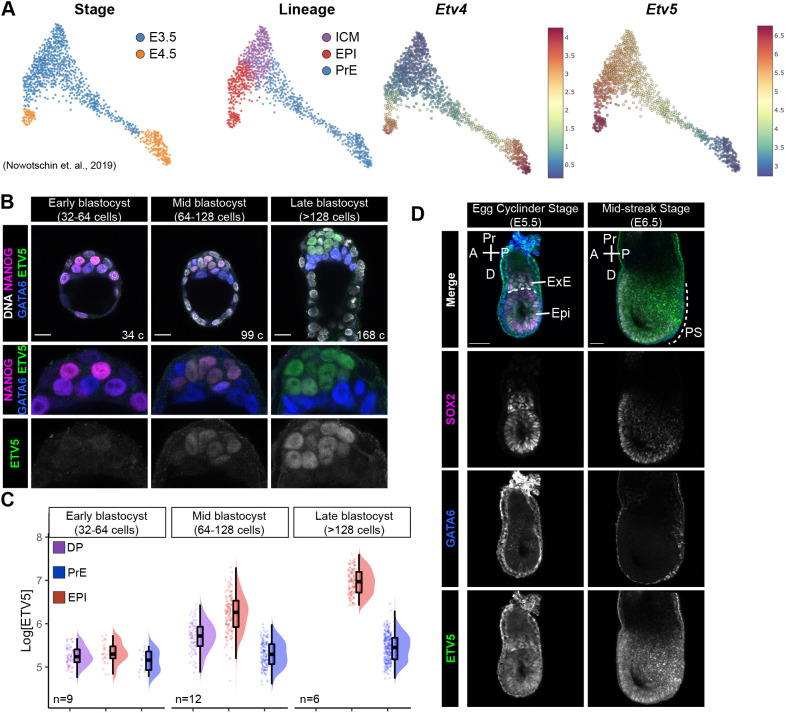
***Etv4* and *Etv5* expression during mouse embryonic development.** (A) Single-cell RNAseq from (Nowotschin et al., 2019) showing *Etv4* and *Etv5* mRNA expression at E3.5 and E4.5 in inner cell mass (ICM), epiblast (EPI) and primitive endoderm (PrE) lineages. (B) Confocal images of immunofluorescence immunostaining of NANOG, GATA6 and ETV5 in blastocyst stage mouse embryos. Cell numbers (c) are indicated. Scale bars: 20 μm. (C) Quantification of ETV5 levels from B. Double positive (DP; NANOG+GATA6), epiblast (EPI; NANOG+) and primitive endoderm (PrE; GATA6+). Sample size: number (*n*) of embryos is indicated. (D) Confocal images of immunofluorescence immunostaining of SOX2, GATA6 and ETV5 in post-implantation stage mouse embryos. Dashed lines indicate the Epi-ExE boundary (E5.5) and the extension of the PS (E6.5). Scale bars: 50 μm. A, anterior; P, posterior; Pr, proximal; D, distal; ExE, extraembryonic-ectoderm; Epi, epiblast; PS, primitive streak.

Given the role of PEA3 family members in the regulation of the pluripotent state in stem cells, we hypothesised that PEA3 family member ETS transcription factors are involved in the establishment of the epiblast lineage. We first characterised where ETV transcripts were expressed during early mouse embryo development using our scRNA-seq dataset from mouse pre- to early post-implantation embryo development (Nowotschin et al., 2019). *Etv4* transcripts were expressed in late-blastocyst stage primitive endoderm (PrE) and epiblast (EPI), whereas *Etv5* transcripts were expressed in early-blastocyst stage inner cell mass (ICM) progenitors, and later in epiblast cells (Fig. 1A).

Next, we assessed protein expression at pre-implantation stages. We were unable to detect the ETV4 protein at pre-implantation stages, likely due to the lack of a good commercially available antibody; however, ETV5 protein was readily detectable. We performed immunofluorescence staining for ETV5, NANOG, GATA6 and quantified nuclear fluorescence intensity to categorise cell lineages (Fig. S1A,B), as described previously (Saiz et al., 2016). In early blastocyst (32-64 cells) stage embryos, ETV5 protein was not expressed. At mid-blastocyst (64-128 cells) stage, low levels of ETV5 protein were detected in uncommitted ICM progenitor cells (NANOG+GATA6+) and higher levels in epiblast cells (NANOG+GATA6-) (Fig. 1B,C). The highest levels of ETV5 protein were detected in the epiblast cells (NANOG+GATA6- or NANOG-GATA6-) of late blastocyst stage embryos (Fig. 1B,C). ETV5 protein was not present in the primitive endoderm (NANOG-GATA6+) or outer trophectoderm cells.

Further to this, we assessed early post-implantation embryos for ETV5 protein expression. We performed immunofluorescence analysis for ETV5, GATA6 (visceral endoderm marker) and SOX2 (epiblast and extra-embryonic ectoderm marker). At the egg cylinder stage (E5.5), ETV5 protein is expressed at low levels within the nuclei of the epiblast and in the distal-most part of the extra-embryonic ectoderm adjacent to the epiblast, and is absent in the visceral endoderm (Fig. 1D). At mid-gastrulation stage (E6.5), ETV5 was expressed heterogeneously in the epiblast and in the nascent mesoderm (Fig. 1D).

Taken together, the transcript and protein profiling reveal that *Etv5* is first expressed in ICM progenitors, and then specifically upregulated in epiblast cells, where expression is maintained in these pluripotent cells through peri- and post-implantation stages.

### Loss of *Etv5* compromises the formation of primitive endoderm

**Fig. 2.**
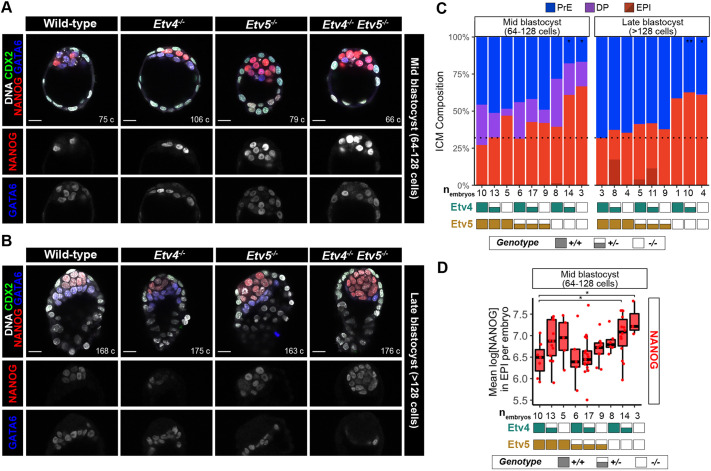
**Loss of *Etv5* compromises the formation of primitive endoderm.** (A,B) Confocal images of immunofluorescence staining of CDX2, NANOG and GATA6 in an *Etv4;Etv5* allelic series of embryos at mid- (A) and late- (B) blastocyst stages. Scale bars: 20 μm. (C) Quantification of inner cell mass (ICM) lineage composition in an allelic series of *Etv4;Etv5* mutant embryos shown in A and B. Double positive (DP; NANOG+GATA6+), primitive endoderm (PrE; NANOG−GATA6+), epiblast (EPI; NANOG+GATA6−, light red; NANOG−GATA6−, dark red). Black dotted line represents wild-type ratio of EPI:PrE at late blastocyst stage. Unpaired *t*-test of PrE numbers compared to wild type: *P<0.05, **P<0.01 (comparisons that are not significant are not indicated). *Etv4^+/+^;Etv5^−/−^* at late-blastocyst stage did not have sufficient *n* values to perform a statistical test. (D) Quantification of NANOG levels in epiblast cells in wild-type embryos and an allelic series of *Etv4;Etv5* mutant embryos shown in A at mid-blastocyst stage. Unpaired *t*-test of mean NANOG levels per embryo compared to wild type: *P<0.05 (comparisons that are not significant are not indicated). Boxplots represent the interquartile range (IQR), with the median shown as a central line; whiskers extend to lowest or highest value within 1.5×IQR; individual data points show mean NANOG levels per embryo.

To determine whether ETVs might act as downstream effectors of FGF signalling in pre-implantation development, we next wanted to assess the role of ETVs in blastocyst formation. Therefore, we analysed cell lineages upon loss of one or both *Etv4* (Livet et al., 2002) and *Etv5* (Zhang et al., 2009) genes . We collected embryos from *Etv4^+/−^;Etv5^+/−^* inter-cross matings at mid- to late blastocyst stage (64- to >128-cell stage), and assessed the number of cells in each lineage by immunofluorescence staining for CDX2 (trophectoderm), NANOG (epiblast) and GATA6 (PrE, Fig. 2A). ICM lineages were assigned by quantification of NANOG/GATA6 levels (Fig. S2A), as described earlier.

From the *Etv4^+/−^;Etv5^+/−^* inter-cross progeny, wild-type embryos and embryos with at least one intact copy of *Etv5*, specified the correct ratio of epiblast cells (Fig. 2A-C), roughly 40% to 60% of PrE (Saiz et al., 2016). Therefore, loss of *Etv4* alone does not alter the epiblast:PrE ratio. Complete loss of *Etv5* (embryos with the genotypes *Etv4^+/+^;Etv5^−/−^*, *Etv4^+/−^;Etv5^−/−^* and *Etv4^−/−^;Etv5^−/−^*), by contrast, had a marked effect on the ratio of epiblast:PrE at both mid-blastocyst and late blastocyst stages (Fig. 2A-C). *Etv5^−/−^* embryos, regardless of *Etv4* genotype, had a reduction in the proportion of primitive endoderm (PrE) cells (57-63% epiblast: 37-43% PrE) by the late blastocyst stage (Fig. 2C). *Etv5^−/^*^−^ embryos exhibited a range in the fraction of primitive endoderm cells in the ICM, with some embryos having fewer percentage of primitive endoderm cells, while others exhibit a complete loss (Fig. 2A,B,  Fig. S2B). The *Etv5^−/−^* embryos phenocopy *Fgfr1^−/−^* embryos (Kang et al., 2017; Molotkov et al., 2017), suggesting a hierarchical relationship of *Fgfr1* and *Etv5* in relaying FGF4 signalling to specify primitive endoderm.

The reduction in the proportion of primitive endoderm cells in *Etv5*-null embryos occurred without changes in total cell counts within the ICM (Fig. S2C). The compensatory increase in epiblast versus primitive endoderm cell numbers in *Etv5*-null embryos suggested that uncommitted cells were preferentially specified to epiblast. The similarity in the number of double-positive cells in single *Etv5*^−/−^, single *Etv4^−/−^*, double *Etv4^−/−^;Etv5^−/−^* knockout and wild-type embryos indicated timely specification of uncommitted progenitors for all embryo genotypes (Fig. 2C). These data, combined with the comparable number of ICM cells between all genotypes (Fig. S2D), argue against a delay in specification or a selective loss of primitive endoderm cells.

Although epiblast specification did not appear to be negatively affected in any of the mutant embryos, we wanted to determine if the epiblast lineage was being properly maintained after specification. Declining NANOG levels are indicative of the exit from naïve pluripotency, as the epiblast undergoes maturation prior to implantation, and is dependent on FGF signalling (Kang et al., 2017; Molotkov et al., 2017; Nichols et al., 2009). NANOG levels were elevated in *Etv4^−/−^* and *Etv5^−/−^* single knockout embryos, when compared with wild-type embryos at the late-blastocyst stage (Fig. 2D). This unphysiologically elevated level of NANOG was further compounded in double knockout embryos (Fig. 2D), with NANOG levels significantly higher in *Etv4^+/−^;Etv5^−/−^* and *Etv4^−/−^;Etv5^−/−^* compared to wild-type embryos, suggesting that both ETV factors play complementary and synergistic roles in the maturation of the epiblast lineage.

All together, these data suggest *Etv5*, but not *Etv4*, is required for balancing the specification of uncommitted ICM cells towards primitive endoderm and away from epiblast cells, to achieve the robust and stereotyped tissue proportions of the blastocyst. However, both *Etv4* and *Etv5* function synergistically to modulate NANOG expression within the epiblast to allow exit from naïve pluripotency.

### Mechanism of *Etv5* action on inner cell mass cell fate decision

**Fig. 3.**
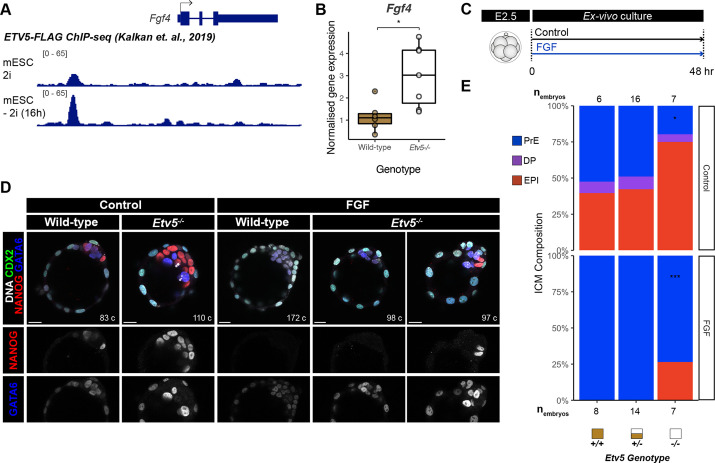
w**Mechanism of *Etv5* action on inner cell mass cell fate decision.** (A) ETV5 binding to the *Fgf4* locus in mouse embryonic stem cells (mESC) in naïve pluripotency conditions (2i) and upon naïve pluripotency exit (16 h after 2i withdrawal). ChIP-seq data are reproduced from Kalkan et al. (2019), where it was published under a CC-BY license (https://creativecommons.org/licenses/by/4.0/). (B) Expression of *Fgf4* in wild-type and *Etv5^−/−^* late-stage blastocyst by qPCR. Unpaired *t*-test: *P<0.05. Boxplots represent the interquartile range (IQR), with the median shown as a central line; whiskers extend to lowest or highest value within 1.5×IQR; individual data points show normalised gene expression levels in each embryo. (C) Schematic of embryo culture treatments. (D) Confocal images of immunofluorescence staining of CDX2, NANOG and GATA6 in wild-type and *Etv5^−/−^* mutant embryos treated with or without 1 μg/ml FGF4+1 μg/ml heparin from E2.5 for 48 h. Scale bars: 20 μm. (E) Quantification of inner cell mass (ICM) lineage composition in treated embryos (C). DP, double positive (NANOG+GATA6+); PrE, primitive endoderm (NANOG−GATA6+); EPI, epiblast (NANOG+GATA6−, red). Unpaired *t*-test: *P<0.05, ***P<0.001.

Given the phenotypic similarity of *Etv5* mutants to mutants with reduced FGF signalling activity (e.g. *Fgf4^+/−^* and *Fgfr1^−/−^* embryos) (Kang et al., 2017, 2013), we reasoned that FGF signalling may be disrupted or dampened in *Etv5* mutant embryos. *Etv5* is expressed at intermediate levels in uncommitted ICM cells and upregulated in epiblast cells, mirroring the expression of *Fgf4* (Fig. 1, Fig. S1B; Nowotschin et al., 2019). Hypothesising that *Etv5* may regulate *Fgf4* transcription, we analysed ChIP-seq data of ETV5 binding (Kalkan et al., 2019) in mouse embryonic stem cells (mESC) cultured in 2i (naïve pluripotent conditions, similar to pre-implantation epiblast) and 16 h after transfer to N2B27 (to induce transition out of the naïve pluripotent state, similar to peri-implantation epiblast). ETV5, a transcriptional activator, is enriched at the upstream promoter region of *Fgf4* in naïve and transitioning mESCs (Fig. 3A), also suggesting direct regulation in epiblast cells *in vivo*.

To test this hypothesis, we analysed *Fgf4* expression after loss of *Etv5* in embryos. Quantitative real-time PCR (qRT-PCR) of E4.5 whole blastocysts demonstrated that *Fgf4* transcripts are elevated in *Etv5^−/−^* embryos compared with wild type (Fig. 3B, fold change=1.59, *P*=0.01). Therefore, in embryos, *Etv5* is dispensable for *Fgf4* expression, which is sustained in *Etv5^−/−^* embryos with higher *Fgf4* expression in *Etv5*^−/−^ embryos correlating with an increased number of epiblast cells. Thus, loss of primitive endoderm in the *Etv5* mutants is not caused by the non-cell autonomous effect of limited FGF4 availability.

Given the dampened response to FGF signalling in the *Etv5^−/−^* embryos, we attempted to rescue the reduction in primitive endoderm numbers with excess exogenous FGF ligand. We treated wild-type, *Etv5^+/−^* and *Etv5^−/−^* embryos with a saturating dose of FGF4 (1 μg/ml) and heparin (1μg/ml) from E2.5 (8-16 cell stage) for 48 h (Fig. 3C). In control culture, wild-type, *Etv5^+/−^* and *Etv5^−/−^* embryos comprised 52%, 49% and 20% GATA6+NANOG− primitive endoderm cells, respectively (Fig. 3D,E and Fig. S3). The significant impairment of primitive endoderm specification in *Etv5^−/−^* control cultured embryos is consistent with freshly recovered *Etv5^−/−^* embryos (Fig. 2). After FGF treatment, wild-type and *Etv5^+/−^* embryos had only GATA6+NANOG− primitive endoderm cells throughout their ICM, as did over half of the *Etv5^−/−^* embryos (4/7 embryos) (Fig. 3D,E and Fig. S3). However, some FGF-treated *Etv5^−/−^* embryos retained GATA6−NANOG+ epiblast cells (3/7 embryos), restoring epiblast ratios to 41%, comparable to untreated wild-type embryos (40% epiblast) (Fig. S3B). These data demonstrate that high, non-physiological levels of exogenous FGF4 can partially rescue primitive endoderm specification in *Etv5^−/−^* mutants, but cannot robustly induce a complete switch in cell fate. Therefore, *Etv5* plays a crucial cell-autonomous role in tuning the sensitivity of ICM cells to the FGF4 signal during primitive endoderm specification.

### Loss of *Etv4/5* causes a delay in the progression of pluripotency

**Fig. 4.**
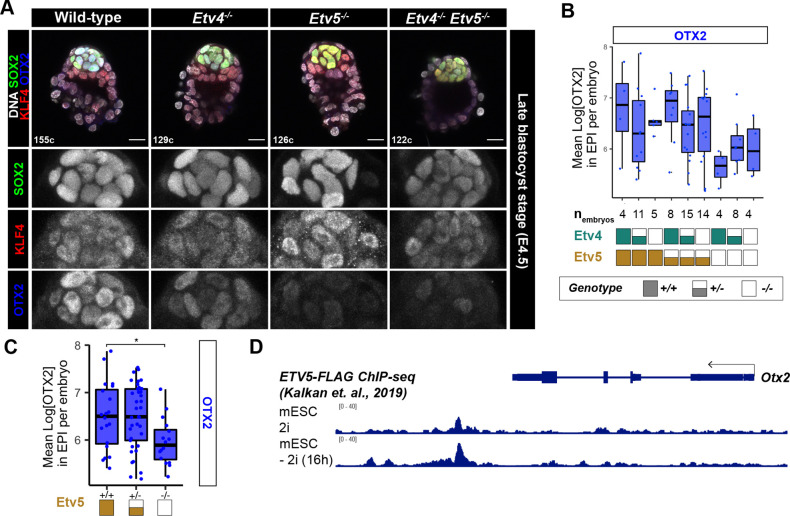
**Loss of *Etv4/5* causes a delay in the progression of pluripotency.** (A) Confocal images of immunofluorescence staining of SOX2, OTX2 and KLF4 in an allelic series of *Etv4;Etv5* mutant embryos at the late blastocyst stage. (B,C) Quantification of primed marker OTX2 from A by individual genotypes (B) or grouped by *Etv5* genotype (C). Boxplots represent the interquartile range (IQR), with the median shown as a central line; whiskers extend to lowest or highest value within 1.5×IQR; individual data points show normalised gene expression levels in each embryo. Unpaired *t*-test of mean OTX2 levels in epiblast cells per embryo, compared with wild type: *P<0.05 (comparisons that are not significant are not indicated). (D) ChIP-seq of ETV5 binding to the *Otx2* locus in mouse naïve ESC (2i) and after pluripotency exit (2i withdrawal). Data reproduced from Kalkan et al. (2019), where it was published under a CC-BY license (https://creativecommons.org/licenses/by/4.0/).

We hypothesised that ETV factors may play a role in epiblast maturation, as suggested by the elevated NANOG levels observed in mutant embryos (Fig. 2D). To probe the role of *Etv4* and *Etv5* in pluripotency exit further, we assessed additional markers of pluripotency at the late blastocyst stage. Embryos from *Etv4^+/−^;Etv5^+/−^* inter-crosses were stained for a core pluripotency marker (SOX2), naïve marker (KLF4) and formative/primed marker (OTX2) (Fig. 4A). While SOX2 and KLF4 in the epiblast were expressed at similar levels across all genotypes (Fig. S4A,B), epiblast OTX2 expression was significantly reduced in embryos lacking *Etv5* (Fig. 4B,C). Analysis of published ETV5 chromatin binding in mESC (Kalkan et al., 2019) shows strong binding at a downstream *Otx2* enhancer region (Fig. 4D). Together, these data indicate that during pre-implantation development, *Etv5* is required for timely exit of pluripotency, likely through direct regulation of *Otx2* and indirect regulation of *Nanog* by an as yet unknown mechanism.

We then assessed pluripotency at early post-implantation stages at E5.5. In wild-type, *Etv4* and *Etv5* null mutants embryos, the epiblast cavitated and retained expression of the core pluripotency factor SOX2 (Fig. S4C). Interestingly, OTX2 was expressed in both the visceral endoderm and the epiblast at this stage, indicating that ETV factors are not necessary for upregulation of primed pluripotency factors at early post-implantation stage.

We then tested the requirements of *Etv4* and *Etv5* for pluripotency in ESCs. We differentiated double knockout *Etv4^−/−^;Etv5^−/−^* ESCs (Lu et al., 2009) to epiblast-like cells (EpiLC) that are representative of the early post-implantation epiblast, which required exogenous FGF2 and activin. After 2 days of EpiLC differentiation, both wild-type and double knockout cells maintained core pluripotency transcription factors (OCT4 and SOX2), downregulated naïve markers (NANOG and KLF4) and upregulated primed markers (Fig. S4D,E). This indicated that loss of *Etv4/5* is not sufficient to completely block naïve pluripotency exit, in agreement with *in vivo* early post-implantation embryo data (Fig. S4C). However, given the failure in downregulation of NANOG and upregulation of OTX2 at pre-implantation stages, ETVs likely control the timely exit of naïve pluripotency in the embryo; this delay has been similarly shown in ESCs (Kalkan et al., 2019).

### Compound *Etv4/5* mutants display developmental delay and anterior visceral endoderm migration defects

**Fig. 5.**
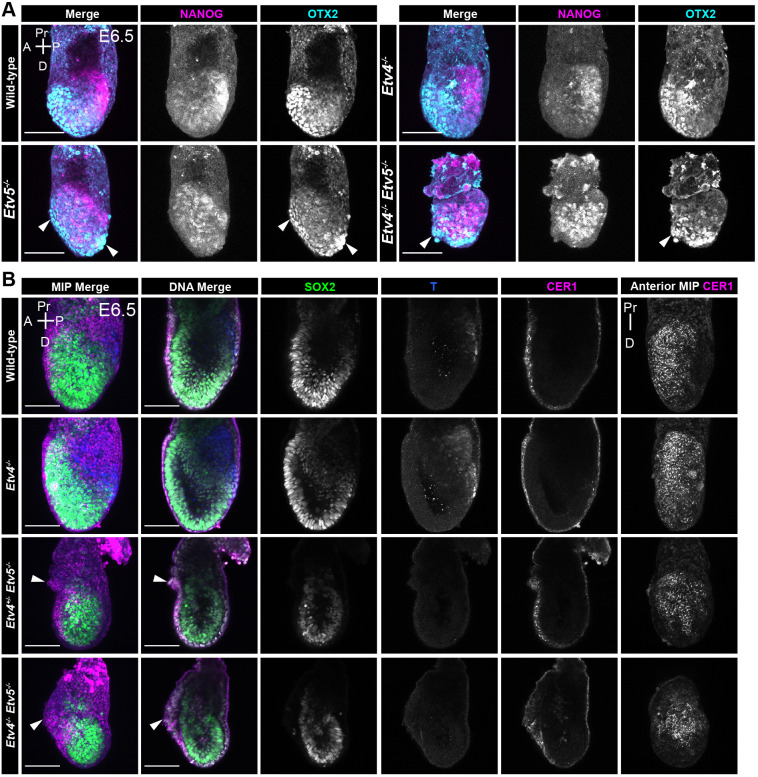
**Compound *Etv4/5* mutants have developmental delay and anterior visceral endoderm migration defects.** (A) Confocal maximum intensity projection images of an allelic series of *Etv4;Etv5* embryos at mid-streak gastrulation stages, embryonic day (E) 6.5, immunostained for NANOG and OTX2. Arrowheads indicate abnormal anterior/distal visceral endoderm migration and/or morphology. (B) Confocal images of an allelic series of *Etv4;Etv5* embryos at mid-streak gastrulation stages, embryonic day (E) 6.5 immunostained for SOX2, T and CER1. Arrowheads indicate abnormal anterior visceral endoderm migration and morphology. MIP, maximum intensity projection; A, anterior; P, posterior; Pr, proximal; D, distal. Scale bars: 100 μm.

We next looked at later embryonic time-points to determine if the delayed naïve pluripotency exit impacted later stages of development. At E6.5, at gastrulation, wild-type and *Etv4* mutant embryos expressed high levels of OTX2 in the anterior visceral endoderm (AVE), and NANOG expression was confined to the proximal posterior region of the epiblast, marking the primitive streak (Fig. 5A). In *Etv5*-null and compound mutants, the distal visceral endoderm (DVE) marked by high OTX2 failed to migrate anteriorly (Fig. 5A, left panel arrowheads). In single *Etv5* mutants, this resulted in a failure to properly position the anterior-posterior axis, as evidenced by the ∼45° rotation of the NANOG expression domain, marking the primitive streak. Double knockout embryos at E6.5 were overall smaller compared to wild-type embryos at the same stage (Fig. 5), and were instead comparable in size to E5.5 wild-type embryos (Fig. S4C).

The extra-embryonic ectoderm region was noticeably reduced in size, with a disordered morphology of the adjacent visceral endoderm epithelium (Fig. 5A, right panel arrowhead). NANOG expression was elevated throughout the epiblast, indicating that either the entire epiblast had failed to exit naïve pluripotency, or the presence of an expanded primitive streak region.

To determine if mutant embryos were specifying the AVE correctly and able to initiate gastrulation, we stained for an AVE marker, CER1, at E6.25 (pre-streak stage) and for a mesoderm marker, T (Brachyury) at E6.5 (mid-streak stage). In wild-type and *Etv4*-null embryos at E6.25 and E6.5, CER1 was localised to the anterior region of the embryo, extending from the embryonic-extraembryonic junction to the distal tip (Fig. 5B, Fig. S5A). At E6.5, T was localised to the posterior epiblast marking the nascent mesoderm (Fig. 5B). However, in single *Etv5* and double *Etv4;Etv5* homozygous mutant embryos at E6.25 and E6.5, while CER1 expression indicated that the AVE had been specified, the migration of the AVE appeared to be disrupted, delayed or arrested at the distal tip of the embryo (Fig. 5B, Fig. S5A, arrowheads). The occasional extension of CER1 expression beyond the embryonic-extraembryonic junction shows an over-migration of the AVE. The CER1 domain size was noticeably reduced relative to the size of the embryos. Again, abnormal thickening and disordered DVE/AVE epithelial morphology were observed in these mutants (Fig. 5B, arrowhead). Finally, at E6.5, mutants were devoid of T+ cells, indicating a failure in gastrulation and primitive streak formation, and/or specification of mesoderm cell types.

The AVE migration phenotype and the reduced embryo size were more severe and pronounced in compound mutant embryos *Etv4^+/−^;Etv5^−/−^* and *Etv4^−/−^;Etv5^−/−^* when compared with single homozygous *Etv4^+/+^;Etv5^−/−^* embryos (Fig. 5A,B, Fig. S5A). Thus, although *Etv4* single mutants did not have an overt phenotype, partial or complete loss of *Etv4* did increase the severity of the *Etv5* phenotype, suggesting that these factors may partially compensate, or have overlapping roles, in the early post-implantation epiblast.

By late-gastrulation (E7.5), *Etv5* and double homozygous null embryos were severely developmentally retarded with a failure in gastrulation, as indicated by the absence of the primitive streak marker T (Fig. S5B). Epiblast morphology was grossly abnormal, with ruffles and folds in the epithelium. As a consequence, *Etv5* homozygous mutant pups were not recovered from *Etv4;Etv5* heterozygous intercrosses, and were drastically under-represented in *Etv5* heterozygous intercrosses (Fig. S5C,D). *Etv4^−/−^;Etv5^+/−^* pups were also not recovered, further suggesting a combinatorial role for ETV factors during embryonic development. All together, these data demonstrate that embryos of *Etv4;Etv5* allelic series have increasingly severe phenotypes with loss of ETV gene dosage at early post-implantation stages exhibiting developmental delay and AVE migration defects that compromise embryonic development.

## DISCUSSION

Here, we explored how transcription factors Etv4 and Etv5 function downstream of FGF signalling to regulate early mouse development. We discovered that Etv5 is essential for primitive endoderm formation before implantation, while the combined loss of Etv4/5 impairs epiblast maturation and proper anterior-posterior patterning after implantation. These findings reveal how FGF signalling effectors orchestrate both early cell fate decisions and subsequent epiblast development necessary for proper gastrulation.

The reduction in the number of primitive endoderm cells in *Etv5^−/−^* blastocyst bears a striking similarity to that of *Fgfr1*^−/−^ and *Fgf4*^+/−^ phenotypes (Brewer et al., 2015; Kang et al., 2017, 2013). Given that *Fgf4* transcripts are not diminished in *Etv5* mutants, the phenocopying of low dosage FGF mutants cannot be due to a decreased ligand availability. Exposure to high levels of exogenous FGF can partially restore primitive endoderm cell numbers in *Etv5*-deficient embryos, suggesting that related ETS family members such as *Etv1* and *Etv4* may compensate for loss of *Etv5* function under these non-physiological conditions, although a complete rescue is not achieved. Therefore, our findings support the hypothesis that *Etv5* primarily relays FGF signalling activity in uncommitted ICM cells to specify primitive endoderm. Here, *Etv5* acts as a FGF signalling effector, similar to other developmental contexts (Herriges et al., 2015; Zhang et al., 2009). The direct involvement of *Etv5* in initiating the primitive endoderm program is further supported by its ability to upregulate multiple endoderm genes (including *Sox7* and *Sox17*) when overexpressed in mouse ESCs (Correa-Cerro et al., 2011).

NANOG, a hallmark marker of mouse naïve pluripotency, is downregulated in the epiblast prior to implantation in an FGF/ERK-dependent manner (Nichols et al., 2009). In both *Etv4^−/−^* and *Etv5^−/−^* blastocysts, elevated NANOG levels reveal a failure to exit naïve pluripotency. *Etv4* and *Etv5* have been implicated in regulating pluripotency *in vitro*. For example, *Etv5* was shown to promote MET during iPSC reprogramming (Zhang et al., 2018). In addition, these ETV factors appear to regulate ESC proliferation, but there is conflicting evidence as to whether they promote or repress epiblast-like fate during differentiation (Akagi et al., 2015; Zhang et al., 2018). Triple knock-out of *Etv5*, *Rbpj* and *Tcf3* in ESCs can maintain naïve pluripotent state in absence of 2i (Kalkan et al., 2019), implicating these factors in dissolution of the naïve pluripotency transcription factor network. Given that ETV5 does not directly bind to the *Nanog* locus in ESCs (Kalkan et al., 2019), it seems likely that this regulation by ETV factors *in vivo* is indirect. Instead, ETVs may activate the primed pluripotency network, which then, in turn, repress the naïve state. Consistent with such a model, the primed pluripotency marker OTX2 fails to be upregulated in *Etv5* mutant blastocysts. ETV5 has been shown to directly bind an *Otx2* enhancer in mESCs (Kalkan et al., 2019), suggesting it is also a direct target *in vivo*. As *Otx2* and *Nanog* are antagonistic (Acampora et al., 2017, 2016), the role of ETV in epiblast cells may be to turn on *Otx2* and consequently reduce *Nanog* levels, thereby promoting the exit from naïve pluripotency.

OTX2, a marker of primed pluripotency, is normally expressed in the epiblast from the late blastocyst stage (E4.5) through early post-implantation. In *Etv5*^−/−^ embryos, however, OTX2 is absent at E4.5, but is eventually expressed by E5.5, indicating a delayed exit from naïve pluripotency. This delay is consistent with our observation of elevated NANOG levels in *Etv5* mutant blastocysts. Furthermore, this is supported by time-course analysis of *Etv5^−/−^* ESC differentiation showing delayed naïve pluripotency exit (Kalkan et al., 2019). Together, these findings highlight a role for the ETVs in regulating the robustness and timely exit of naïve pluripotency.

Timely Nodal-dependent maturation of the epiblast at early post-implantation stages is required for the coordinated and spatial patterning of the embryo and initiation of gastrulation (Huang et al., 2017; Zang et al., 2022). Given that *Etv4* and *Etv5* are not expressed in the visceral endoderm, we hypothesize that the delayed epiblast maturation observed in *Etv5* and *Etv4;Etv5* compound mutants leads to the later AVE defects. In the *Etv5* and *Etv4;Etv5* compound mutants, there is a reduction in the number of CER1-expressing cells, disordered discontinuous AVE and ectopic protrusions. These phenotypes are reminiscent of mutants affecting AVE development, including mutants in Nodal signalling pathway components (Stower and Srinivas, 2014). The most severely affected *Etv4^−/−^;Etv5^−/−^* embryos either fail to migrate the AVE, or the AVE over-migrates beyond the embryonic-extraembryonic boundary, and exhibit a disordered multi-layered epithelium, reminiscent of the two classes of *Lefty1* mutants (a Nodal antagonist) (Trichas et al., 2011). Our research suggests that FGF, in conjunction with Nodal, plays a role in epiblast development and its interaction with the visceral endoderm, which is crucial for establishing the anterior-posterior axis of the embryo. All together, our results demonstrate the successive roles of ETS factors *Etv4* and *Etv5* as FGF signalling effectors in early lineage specification and embryonic body plan establishment, increasing our understanding of the molecular mechanisms of mammalian development.

## MATERIALS AND METHODS

### Immunofluorescence

Pre-implantation embryo immunofluorescence was carried out as previously described (Saiz et al., 2016). Briefly, embryos were fixed for 10 min at room temperature in 4% PFA. Fixed blastocysts were washed in PBX [0.1% Triton X-100 (Sigma-Aldrich) in PBS], permeabilised for 5 min in a solution of 0.5% Triton X-100 and 100 mM glycine in PBS, and then washed in PBX for 5 min. Embryos were blocked in blocking buffer (2% horse serum in PBS) for 40 min at room temperature, followed by incubation overnight at 4°C with primary antibodies diluted in blocking buffer (Table S1). The next day, embryos were washed three times in PBX, incubated in blocking buffer for 40 min at room temperature before a 1 h incubation with secondary antibodies at 4°C. Embryos were then washed in PBX and incubated in 5 μg/ml Hoechst in PBS for at least 30 min prior to imaging.

For post-implantation stages, embryos were fixed for 30 min at room temperature in 4% PFA. Then, fixed embryos were washed in PBX, permeabilised in 0.5% Triton-X in PBS for 30 min and then washed three times in PBX. Embryos were then incubated in blocking buffer, 5% donkey serum and 0.2% BSA in PBX for 2 h at room temperature, followed by incubation overnight at 4°C with primary antibodies diluted in blocking buffer. The next day, embryos were washed in PBX, followed by a second blocking step for at least 2 h at room temperature, and incubation with secondary antibodies in blocking buffer overnight at 4°C. After antibody staining, embryos were washed in PBX, and incubated with 5 μg/ml Hoechst in PBX for a minimum of 2 h to visualise DNA prior to imaging.

### Image acquisition and analysis

Embryos were imaged on a Zeiss LSM880 laser scanning confocal microscope, in glass bottomed dishes (MakTek) in PBS. Pre-implantation embryos were imaged using a Plan-Neofluar 40×/1.30 oil immersion objective. Post-implantation embryos were imaged at 20× using a Pan-Apo 20×/0.8 air objective. Nuclear segmentation for quantification of fluorescence intensity was carried out using MINS, as described previously (Lou et al., 2014). Correction for fluorescence decay along the *z*-axis was performed by linear regression and empirical Bayes method, as detailed by Saiz et al. (2016).

### Cell lines

ESC lines used in the study were wild-type and *Etv4^−/−^;Etv5^−/−^* cells (Lu et al., 2009). ESCs were maintained on 0.1% gelatin (Millipore, 104070) coated tissue-culture grade plates in a humidified 37°C incubator with 5% CO_2_. ESCs were grown in DMEM (Life Technologies, 11995073), supplemented with 2 mM L-glutamine (Life Technologies, 25030164), 0.1 mM MEM NEAA (Life Technologies, 11140-050), 1 mM sodium pyruvate (Life Technologies, 11360070), 100 U/ml penicillin and 100 mg/ml streptomycin (Life Technologies, 15140163), 0.1 mM 2-mercaptoethanol (Life Technologies, 21985023), 15% FBS (VWR, 97068-085) and 1000 U/ml LIF (prepared in house). ESCs were differentiated to EpiLCs as previously described (Hayashi et al., 2011). Briefly, ESCs were seeded 2.5×10^4^ cells/cm^2^ in EpiLC medium onto fibronectin-coated (16 μg/ml, Millipore, FC010) 8-well IBIDI plates. EpiLC medium comprised N2B27 medium containing 20 ng/ml activin A (Peprotech, 120-14P), 12 ng/ml FGF2 (R&D Systems, 233-FB-025) and 1% KSR (ThermoFisher Scientific, 10828028).

### Mice and genotyping

Animal work was approved by the MSKCC Institutional Animal Care and Use Committee (IACUC). *Etv4^tm1Arbr^* (Livet et al., 2002) and *Etv5^tm1.1Xsun^* (Zhang et al., 2009) knockout alleles (abbreviated to *Etv4^−^* or *Etv5^−^*) were maintained as double and single heterozygous mouse lines on a CD1 background. Mice, whole blastocysts, whole embryos or trophectoderm cells were genotyped by PCR. Three primer PCRs were used to genotype *Etv5* samples, as follows: F, 5′-CTCGCAGAGGACAAGGTAGTGAC-3′; R_WT, 5′-GTGTGCACGACATGTTCAAGG-3′; and R_KO, 5′- CCAGCATCGTACAAAACAAGAG-3′. These generated a wild-type band at 270 bp and a knockout band at 374 bp. For genotyping *Etv4* samples, the following primers were used: Etv4_WT_F (5′-TCTGGACCCTCTCCAGGTGATG-3′) and Etv4_WT_R (5′-CCACCAGAAACTGCCACAGTTG-3′), generating a wild-type band of 501 bp; and LacZ_F (5′-CATCCACGCGCGCGTACATC-3′) and LacZ_R (5′-CCGAACCATCCGCTGTGGTAC-3′), generating a knockout band, amplifying the LacZ cassette of 360 bp.

### FGF embryo treatment

Embryos for this study were obtained from natural matings between *Etv5* male and female heterozygotes. The sex of embryos was not determined. E2.5 morulae were flushed from oviducts with flushing holding medium (FHM, Millipore) as described previously (Behringer et al., 2014). Embryos within litters were randomly assigned in even-sized groups for control and exogenous FGF treatment: control, KSOM (MR-121-D, Sigma); FGF stimulation, 1 μg/ml FGF4 (R&D Systems) and 1 μg/ml heparin (Sigma) in KSOM. Medium was equilibrated 30 min prior to culture to reach the correct temperature and pH. Embryos were cultured in groups in droplets of medium in 35 mm dishes (∼1 μl/embryo) overlaid with mineral oil (Sigma) for 48 h in total in a humidified incubator at 37°C with 5% CO_2_. After 24 h (E3.5), the zona pellucidae were removed by brief incubation in acid Tyrode's solution (Sigma), washed three times in their respective culture medium and then cultured in fresh droplets of medium. Embryos were assayed for lineage markers by immunofluorescence at the end of a 48 h culture.

### Single-embryo qPCR

E4.5 stage embryos from *Etv5* intercrosses were prepared for genotyping and qPCR as previously described (Kang et al., 2017; Morgani et al., 2018), using CellsDirect One-Step kit in accordance with the manufacturer's instructions. Blastocyst were first washed in PBS, then incubated in 0.5% trypsin for 3 min at 37°C. Using a glass capillary, a small number of mural TE cells were removed for PCR genotyping, then each blastocyst was added to 5 µl of 2×Reaction Mix (Invitrogen, CellsDirect One-Step qRT-PCR Kit), snap-frozen on dry ice and stored at −80°C until processing.

For cDNA and target-specific pre-amplification, 5 μl of a reverse transcription/pre-amp mix was added to each blastocyst lysate. For each sample this comprised 0.2 μl SuperScript II RT/Platinum Taq mix (Invitrogen), 2.5 μl TaqMan assay (pooled assay mix with a concentration of 0.2× for each probe, detailed in Table S2), 2.3 μl RNase-free H_2_O. To perform combined cell lysis, cDNA synthesis and pre-amplification of specific targets, samples were incubated at 50°C for 20 min, 95°C for 2 min, followed by 18 cycles of 95°C for 15 s then 60°C for 4 min, in a T100 Thermal Cycler (Bio-Rad).

Blastocyst cDNA was diluted 1 in 5 by adding 40 μl H_2_O to the 10 μl cDNA to a total of 50 μl. To assay the amount of mRNA, each qPCR reaction was set up in duplicate in 96-well plates (Applied Biosystems, 4306737) overlaid with MicroAmp clear adhesive film (Applied Biosystems, 4306311). For each qPCR reaction, the mix was as follows: 7.5 μl TaqMan Universal PCR Master Mix, 0.75 μl TaqMan Assay and 5.25 μl RNase free H_2_O. Sample cDNA (1.5 μl diluted) was then added to each reaction, to a total of 15 μl. Real-time PCR was then carried out in a QuantStudio 7 Flex System (Applied Biosystems). Gene expression was calculated as 2^ΔΔCt^. Target gene (FGF) expression was calibrated to the arithmetic mean of the wild-type samples, and normalised to the expression of two reference genes within each sample, *Gapdh* and *Actb*, using the geometric mean (Vandesompele et al., 2002).

## Supplementary Material



10.1242/develop.204278_sup1 Supplementary information
