# Supplementary material for: ETV4 and ETV5 orchestrate FGF-mediated lineage specification and epiblast maturation during early mouse development
Source: Development. 2025 Mar 24;152(6):dev204278. doi: 10.1242/dev.204278 (PMC12050069; doi:10.1242/dev.204278)
Supplement: Supplementary information [file develop-152-204278-s1.pdf]

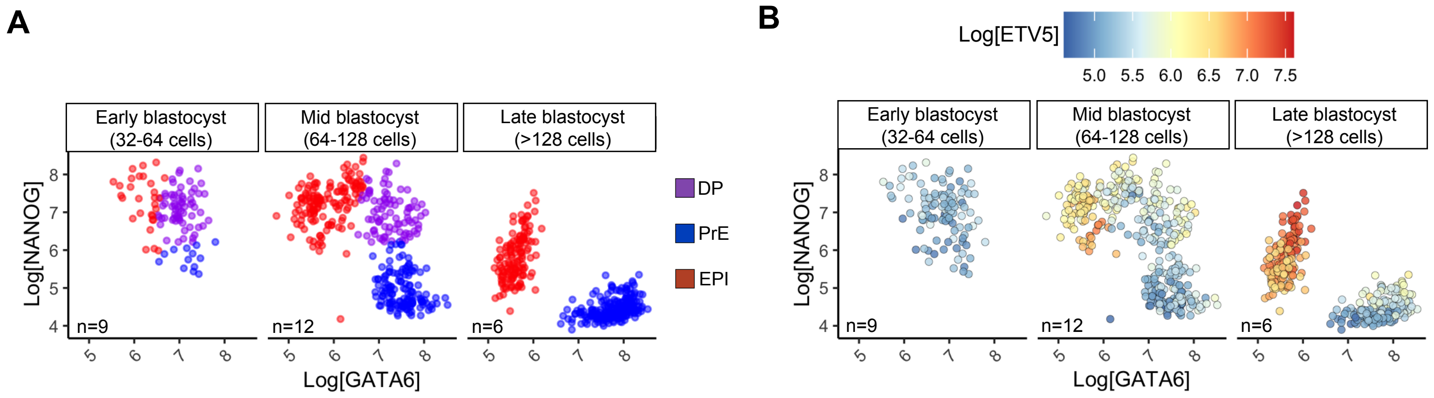

Fig. S1. *Etv4* and *Etv5* expression during mouse embryonic development (related to Figure 1)

(A) Quantification of NANOG and GATA6 levels in blastocyst (from Figure 1B). Clustering into double positive (DP; NANOG+;GATA6+), epiblast (EPI; NANOG+;GATA6-) and primitive endoderm (PrE; NANOG-;GATA6+)

(B) Heatmap of ETV5 levels in blastocyst compared to NANOG and GATA6 levels (from Figure 1B)

Development • Supplementary information

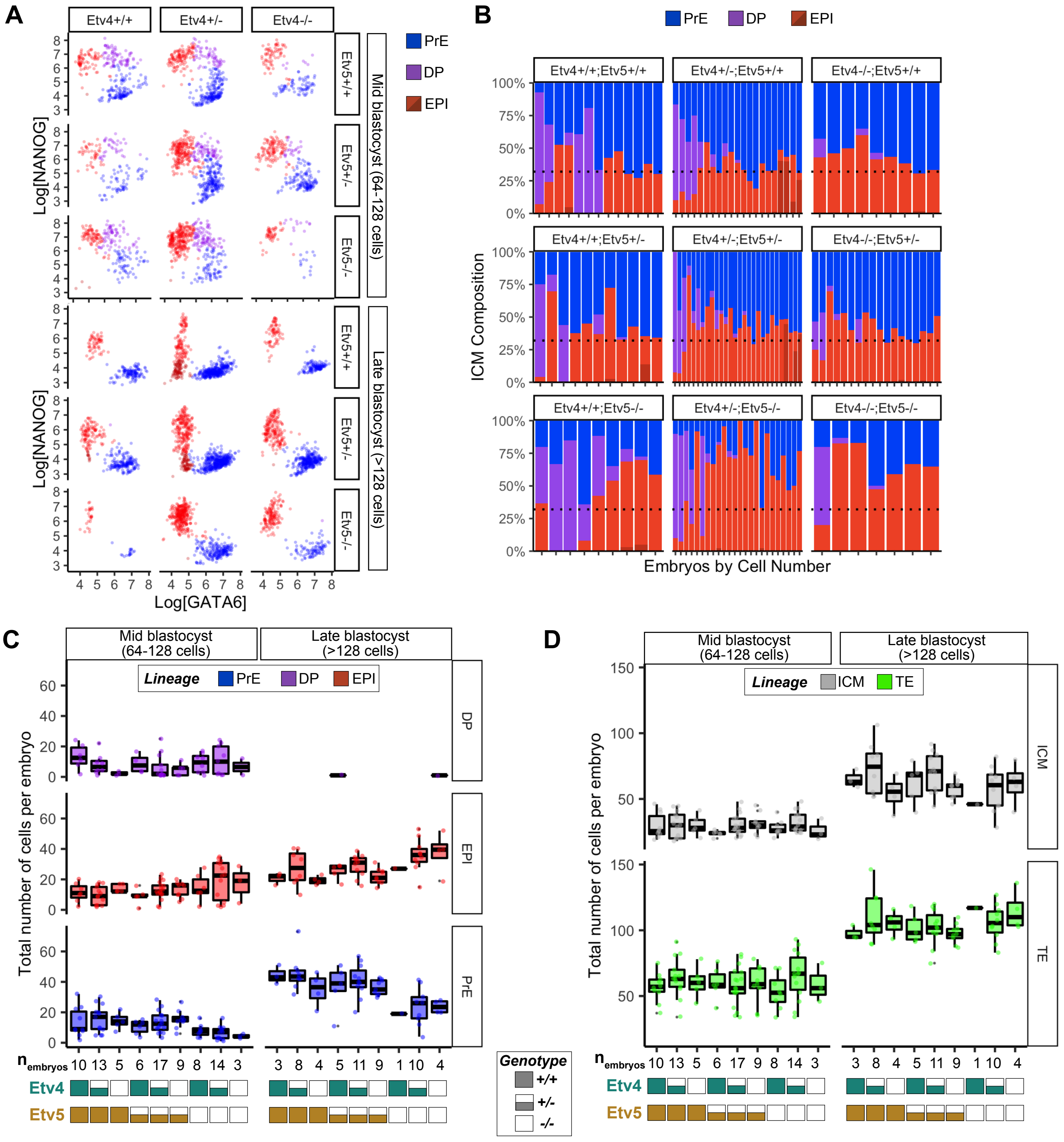

Fig. S2. Loss of *Etv5* compromises the formation of PrE (related to Fig. 2)

(A) Quantification of NANOG and GATA6 levels in an *Etv4;Etv5* allelic series of mutant embryos (from Figure 2A,B). Clustering into double positive (DP; NANOG+;GATA6+, purple), primitive endoderm (PrE; NANOG-;GATA6+, blue), epiblast (EPI; NANOG+;GATA6- light red, NANOG-;GATA6- dark red).

(B) Quantification of inner cell mass (ICM) lineage composition in an allelic series of *Etv4;Etv5* mutant embryos. Individual embryos shown and ordered by ascending cell number. Dotted line represents mean wild-type EPI:PrE composition by late blastocyst stage. (DP: purple, PrE: blue, EPI: red)

(C) Total number of cells per embryo in DP (purple), EPI (red) and PrE (blue) lineages

(D) Total number of cells per embryo in ICM (grey) and TE (green) lineages.

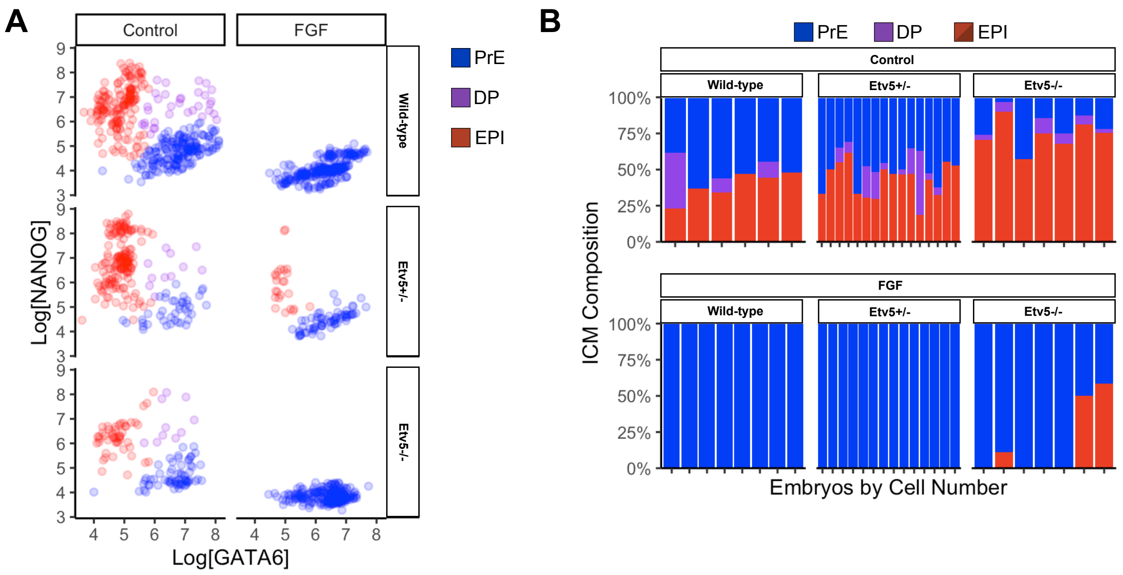

Fig. S3. Mechanism of *Etv5* action on ICM cell fate decision (related to Fig. 3)

(A) Quantification of NANOG and GATA6 levels in control and FGF treated wild-type, *Etv5*$^{+/-}$ and *Etv5*$^{-/-}$ embryos (related to Figure 3B). Clustering into primitive endoderm (PrE; blue), epiblast (EPI; red), and double positive (DP; purple).

(B) Quantification of inner cell mass (ICM) lineage composition in control and FGF treated wild-type, *Etv5*$^{+/-}$ and *Etv5*$^{-/-}$ embryos (PrE: blue, EPI: red, DP: purple). Individual embryos shown and ordered by ascending cell number.

Development • Supplementary information

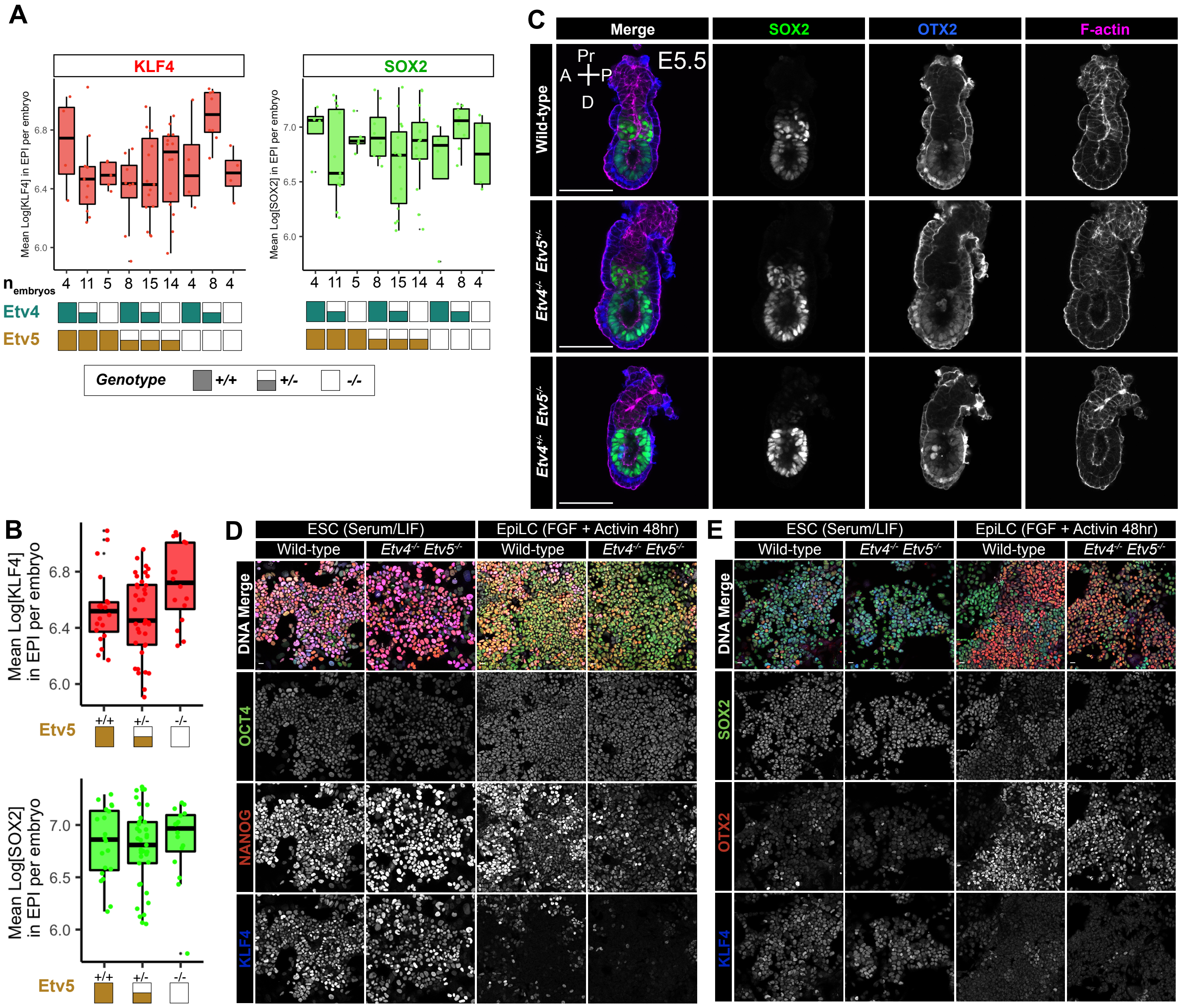

Fig. S4. Loss of *Etv4/5* causes a delay in the progression of pluripotency

(A) Quantification of KLF4 and SOX2 levels in epiblast cells per embryo in an allelic *Etv4;Etv5* series of late-stage blastocysts (from Figure 4A).

(B) Quantification of KLF4 and SOX2 levels in epiblast cells per embryo in an allelic *Etv4;Etv5* series of late-stage blastocysts from (A) grouped by *Etv5* genotype.

(C) Confocal images of immunofluorescence staining of SOX2, OTX2 and F-actin in post-implantation *Etv4;Etv5* day E5.5. embryos. Pr = Proximal, D = Distal, A = Anterior, P = Posterior. Scale bar 100μm

(D-E) Confocal images of immunostaining for OCT4, NANOG, KLF4 (D) and SOX2, OTX2, KLF4 (E) in wild-type and *Etv4*$^{-/-}$;*Etv5*$^{-/-}$ mESC in naïve conditions (Serum/LIF) and differentiated to epi-like cells (EpiLC, FGF+Activin 48h). Scale bar 20μm

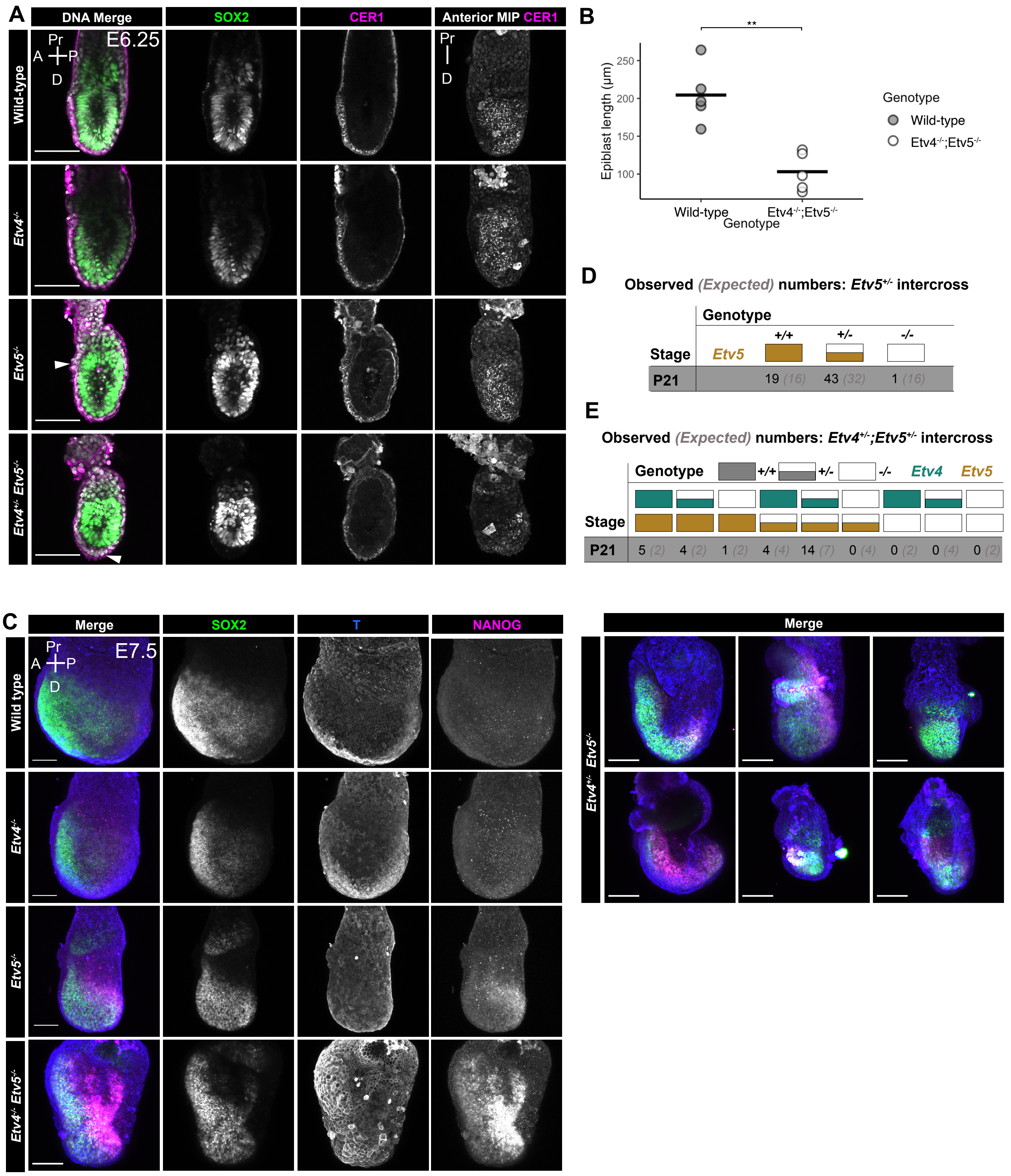

Fig. S5. Compound *Etv4/5* mutants have developmental delay and anterior visceral endoderm migration defects

(A) Confocal images of an allelic series of *Etv4;Etv5* embryos at pre-streak stages, embryonic day (E) 6.25 immunostained for SOX2, and CER1. Anterior max intensity projection (right) and z-slice (left). Arrow heads show abnormal anterior/distal visceral endoderm migration and/or morphology. A = Anterior, P= Posterior. Pr = Proximal. D = Distal. Scale bar 100µm

(B) Confocal maximum intensity projection images of an allelic series of *Etv4;Etv5* embryos at late-bug gastrulation stages, embryonic day (E) 7.5 immunostained for SOX2, T and NANOG. Arrow head shows abnormal anterior/distal visceral endoderm migration and/or morphology. A = Anterior, P= Posterior. Pr = Proximal. D = Distal. Scale bar 100µm. Range of abnormal morphologies amongst *Etv4$^{+/-}$;Etv5$^{-/-}$* embryos (right).

(C-D) Observed and expected numbers from *Etv5$^{+/-}$* **(C)** and *Etv4$^{+/-}$;Etv5$^{+/-}$* **(D)** heterozygous intercrosses at weaning, postnatal day (P) 21.

Development • Supplementary information

Table S1. Antibodies used in this study

Antibody	Species	Company	Catalogue #	RRID	Dilution
Anti-Etv5	Rabbit	Proteintech	13011-1-AP	AB_2278092	1:100
Anti-Gata6	Goat	R&D	AF1700	AB_2108901	1:100
Anti-Nanog	Rabbit	Reprocell	REC-RCAB0002PF	AB_567471	1:500
Anti-Nanog	Rat	e-biosciences	14-5761	AB_2865129	1:100
Anti-Sox2	Rat	e-biosciences	14-9811-82	AB_11219471	1:200
Anti-Klf4	Rabbit	Cell Signalling	4038	AB_2265207	1:100
Anti-Otx2	Goat	R&D	AF1979	AB_2157172	1:500
Anti-Cer1	Goat	R&D	AF1986	AB_2077219	1:300
Anti-T	Goat	R&D	AF2085	AB_2200235	1:100
Anti-T	Rabbit	Abcam	ab209665	AB_2750925	1:100

Table S2. TaqMan Gene Expression Assays used in this study

Gene	Assay ID
Actb	Mm00607939_s1
Fgf4	Mm00438916_g1
Gapdh	Mm99999915_g1
