# Supplementary material for: ETV4 and ETV5 orchestrate FGF-mediated lineage specification and epiblast maturation during early mouse development
Source: Development. 2025 Mar 24;152(6):dev204278. doi: 10.1242/dev.204278 (PMC12050069; doi:10.1242/dev.204278)
Supplement: Supplementary file 1 [file develop-152-204278_review_history.pdf]

ETV4 and ETV5 orchestrate FGF mediated lineage specification and epiblast maturation during early mouse development

Claire Simon, Woonyung Hur, Vidur Garg, Ying-Yi Kuo, Kathy K. Niakan, Anna-Katerina Hadjantonakis
DOI: 10.1242/dev.204278

Editor: Liz Robertson

Review timeline

Original submission:	25 July 2024
Editorial decision:	20 August 2024
First revision received:	31 January 2025
Accepted:	4 February 2025

Original submission

First decision letter

MS ID#: dev.204278

MS Title: ETV4 and ETV5 Orchestrate FGF Mediated Lineage Specification and Epiblast Maturation during Early Mouse Development

Authors: Claire Simon; Vidur Garg; Ying-Yi Kuo; Kathy K. Niakan; Anna-Katerina Hadjantonakis
Article Type: Research Article

I have now received all the referees reports on the above manuscript, and have reached a decision. The referees' comments are appended below, or you can access them online: please go to.

The overall evaluation is positive and we would like to publish a revised manuscript in Development, provided that the referees' comments can be satisfactorily addressed. Please attend to all of the reviewers' comments in your revised manuscript and detail them in your point-by-point response. If you do not agree with any of their criticisms or suggestions explain clearly why this is so. If it would be helpful, you are welcome to contact us to discuss your revision in greater detail. Please send us a point-by-point response indicating your plans for addressing the referees' comments, and we will look over this and provide further guidance.

Comments from the Reviewers:

Reviewer 1: SUMMARY OF THE ADVANCE MADE IN THIS PAPER AND ITS POTENTIAL SIGNIFICANCE TO THE FIELD

In this manuscript Simon and co-authors investigate the roles of the factors ETV4 and ETV5 in early mouse development and exit from naïve pluripotency. They generate compound mutant embryos to take account of the potential overlap in activity of the two factors. This work was partly inspired by the group's previous work investigating the roles of FGF receptors during early mouse development, and partly by previous studies characterising the roles of these and other factors during exit from pluripotency in vitro. Here, they make use of compound mutant embryos produced by mating ETV4+/-; ETV5+/- mice. They observe a reduction in the proportion of primitive endoderm, particularly in ETV5 null embryos, but not total ICM cell number, implying reduced induction of PrE, and leading to defects in visceral endoderm differentiation, particularly affecting DVE/AVE function. Interestingly, PrE can be induced to differentiate by adding FGF4 to the early

© 2025. Published by The Company of Biologists under the terms of the Creative Commons Attribution License (https://creativecommons.org/licenses/by/4.0/).

embryo culture. The mutant embryos also exhibit a delay in maturation of peri-implantation epiblast causing maintenance of high levels of Nanog, which is exacerbated in compound ETV4 and 5 mutants. Immunohistochemistry revealed that the pluripotency factors Sox2 and Klf4 were expressed normally in mutant embryos, but Otx2 was reduced. Postimplantation, ETV5-/- embryos failed to undergo DVE anterior migration; the primitive streak could not form normally and the level of Nanog did not become reduced in the anterior epiblast. Cer1 was abnormally expressed in the compound mutant embryos. Not surprisingly, no ETV5-/- pups were born.

SUGGESTIONS TO AUTHORS
The work is clearly and logically presented with detailed reference to previous work leading up to this project. The figures are excellent and not over-complicated. My only request is that the authors check Supp Fig 4C, in which the bottom panel is labelled as ETV4+/-; ETV5-/-. I suspect this should be double the negative embryo. It is interesting because the extraembryonic ectoderm is not expressing Sox2, which would suggest loss of FGF4 from the epiblast. Please could the authors comment on this phenotype and the frequency with which it is observed?

Reviewer 2: SUMMARY OF THE ADVANCE MADE IN THIS PAPER AND ITS POTENTIAL SIGNIFICANCE TO THE FIELD
This manuscript provides an essential insight on the regulation of the naïve - primed pluripotency transition by Etv5, as well as on the role of Etv5 and 4 in the primitive endoderm formation. Importantly, it provides evidence that the delayed pluripotency exit may lead to anomalies in anterior-posterior axis formation and failure in gastrulation.
It is an important piece of work that would benefit the scientific community working on pluripotency and early mammalian development.

SUGGESTIONS TO AUTHORS

The work included here is convincingly presented and I have only a few major and several minor comments and suggestions on how to improve the manuscript.

Major comments:

1. It is not clear to me why in supplementary fig 3A, in the wt group control, two embryos did not form any epiblast cells and several others have a mixture of uncommitted and primitive endoderm cells. Similarly, in the same experiment, two of the Etv5+/- embryos that were not treated with FGF had also 100% of their ICM cells committed to primitive endoderm while the other embryos from this group had very low percentage of epiblast cells when comparing to the freshly flushed Etv5+/- Etv4+/+. This is highly unusual and may suggest some kind of contamination between FGF treated embryos and the wt control group. Another potential explanation is that culture conditions somehow influenced/compromised the development of those embryos. Either way I strongly suggest that the authors repeat this experiment while making sure that the control embryos do not have any contact with FGF or provide a compelling explanation for the observed discrepancy between the results from freshly flushed and control embryos. Alternatively, the authors may choose to remove these data from the manuscript as they do not add much to the overall picture.

2. In the whole paragraph starting from line 259 the authors should include the stage of the embryos they refer to as they seem to discuss both the data from E6.5 and E 6.25 embryos but this is not clear from the text.

3. In the paragraph starting from line 274, could the authors indicate which figure/s they refer to?

Minor points:

Fig 1C. I am assuming nr in the graph are the nr of embryos analysed. Please add explanation to the figure legend

Fig 1 in general - I believe that supplementary fig 1C should be moved to the main fig 1. It is not clear why Fig 1 contains only pre/peri implantation stages

© 2025. Published by The Company of Biologists under the terms of the Creative Commons Attribution License (https://creativecommons.org/licenses/by/4.0/).

Supplementary Fig 2 D - colour code needs to be provided. Also, I am assuming that colour code is the same for section A-C of this figure, but this is not explicitly stated in the figure legend

Line 128; Sentence: "We collected embryos from Etv4+/-;Etv5+/- inter-cross matings at early to late blastocyst stage, and assessed the number of cells in each lineage by quantitative immunofluorescence staining for CDX2 (trophectoderm), NANOG (epiblast) and GATA6 (PrE, Supplemental Fig. 2A) as described earlier." - Supplementary fig 2A does not show TE cells

Line 138, 141,142 - The authors refer to the wrong figure. Please correct

Line 159 and 173; primitive endoerm should be corrected to primitive endoderm

Line 217-8; sentence: "Analysis of published Etv5 chromatin binding in mESC (Kalkan et al., 2019) shows strong binding at a downstream enhancer region (Fig. 4D)." refers to Otx2 but it may be worth to spell it out.

It would be a good idea to include the stage of the embryo in the supplementary figure 5 B, to make it consistent with other figures.

Line 252-3; sentence: "Double knockout embryos at E6.5 were overall smaller compared to E5.5 wild-type embryos." Is the E5.5 a typo? If not, can the authors provide any data that show the comparison between double mutant E6.5 and wt E5.5 embryos?

Line 379; "After antibody staiing," it should be "staining

First revision

Author response to reviewers' comments

Comments from the Reviewers:

Reviewer 1: SUMMARY OF THE ADVANCE MADE IN THIS PAPER AND ITS POTENTIAL SIGNIFICANCE TO THE FIELD

In this manuscript Simon and co-authors investigate the roles of the factors ETV4 and ETV5 in early mouse development and exit from naïve pluripotency. They generate compound mutant embryos to take account of the potential overlap in activity of the two factors. This work was partly inspired by the group's previous work investigating the roles of FGF receptors during early mouse development, and partly by previous studies characterising the roles of these and other factors during exit from pluripotency in vitro. Here, they make use of compound mutant embryos produced by mating ETV4+/-; ETV5+/- mice. They observe a reduction in the proportion of primitive endoderm, particularly in ETV5 null embryos, but not total ICM cell number, implying reduced induction of PrE, and leading to defects in visceral endoderm differentiation, particularly affecting DVE/AVE function. Interestingly, PrE can be induced to differentiate by adding FGF4 to the early embryo culture. The mutant embryos also exhibit a delay in maturation of peri-implantation epiblast causing maintenance of high levels of Nanog, which is exacerbated in compound ETV4 and 5 mutants. Immunohistochemistry revealed that the pluripotency factors Sox2 and Klf4 were expressed normally in mutant embryos, but Otx2 was reduced. Postimplantation, ETV5-/- embryos failed to undergo DVE anterior migration; the primitive streak could not form normally and the level of Nanog did not become reduced in the anterior epiblast. Cer1 was abnormally expressed in the compound mutant embryos. Not surprisingly, no ETV5-/- pups were born.

SUGGESTIONS TO AUTHORS

The work is clearly and logically presented with detailed reference to previous work leading up to this project. The figures are excellent and not over-complicated. My only request is that the

© 2025. Published by The Company of Biologists under the terms of the Creative Commons Attribution License (https://creativecommons.org/licenses/by/4.0/).

authors check Supp Fig 4C, in which the bottom panel is labelled as ETV4+/-; ETV5-/-. I suspect this should be double the negative embryo. It is interesting because the extraembryonic ectoderm is not expressing Sox2, which would suggest loss of FGF4 from the epiblast. Please could the authors comment on this phenotype and the frequency with which it is observed?

The reviewer is right, the low levels of Sox2 in the extraembryonic ectoderm is a very interesting observation. The localisation of Sox2 in the ExE likely corresponds to the trophoblast stem cells, which are dependent on FGF signalling for their proliferation in vivo and in vitro. The labelling of the genotyping in Supp Fig 4C is correct, as we didn't recover Etv4;Etv5 double homozygous embryos in these E5.5 experiments.

We find that the Sox2 expression in the ExE is highly dynamic at this stage of development. For example, in younger E5.5 embryos within the same litters as those shown in Supp Fig 4C (Rebuttal Fig. 1), there were examples of single heterozygotes, which behave like wild-types, that also had a reduction of Sox2 in the ExE. Suggesting that levels of Sox2 in the ExE are rapidly upregulated at this stage. Unfortunately, due to our small sample size at E5.5 we cannot draw definitive conclusions about the levels of Sox2 in the mutants compared to wild-type, as this would require a large enough cohort to perform appropriate stage matching. At E6.5, in both Etv5 single homozygous, and Etv4;Etv5 double homozygous embryos, Sox2 expression in the ExE is much stronger and comparable to wild-type embryos at the same stage (Fig. 5). For these reasons, we have not referred to differences in Sox2 in the ExE in the main text.

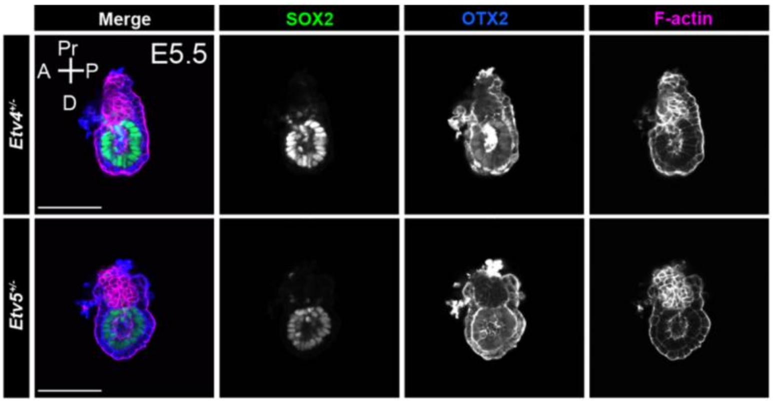

Rebuttal Fig. 1
Confocal images of immunofluorescence staining of SOX2, OTX2 and F-actin in post- implantation day E5.5. embryos. Pr = Proximal, D = Distal, A = Anterior, P = Posterior. Scale bar 100µm

Reviewer 2: SUMMARY OF THE ADVANCE MADE IN THIS PAPER AND ITS POTENTIAL SIGNIFICANCE TO THE FIELD

This manuscript provides an essential insight on the regulation of the naïve – primed pluripotency transition by Etv5, as well as on the role of Etv5 and 4 in the primitive endoderm formation. Importantly, it provides evidence that the delayed pluripotency exit may lead to anomalies in anterior-posterior axis formation and failure in gastrulation.
It is an important piece of work that would benefit the scientific community working on pluripotency and early mammalian development.

SUGGESTIONS TO AUTHORS

The work included here is convincingly presented and I have only a few major and several minor comments and suggestions on how to improve the manuscript.

Major comments:

1. It is not clear to me why in supplementary fig 3A, in the wt group control, two embryos did

© 2025. Published by The Company of Biologists under the terms of the Creative Commons Attribution License (https://creativecommons.org/licenses/by/4.0/).

not form any epiblast cells and several others have a mixture of uncommitted and primitive endoderm cells. Similarly, in the same experiment, two of the Etv5+/- embryos that were not treated with FGF had also 100% of their ICM cells committed to primitive endoderm while the other embryos from this group had very low percentage of epiblast cells when comparing to the freshly flushed Etv5+/- Etv4+/+. This is highly unusual and may suggest some kind of contamination between FGF treated embryos and the wt control group. Another potential explanation is that culture conditions somehow influenced/compromised the development of those embryos.

Either way I strongly suggest that the authors repeat this experiment while making sure that the control embryos do not have any contact with FGF or provide a compelling explanation for the observed discrepancy between the results from freshly flushed and control embryos. Alternatively, the authors may choose to remove these data from the manuscript as they do not add much to the overall picture.

We have repeated the Control and FGF treatments of Wild-type, Etv5+/- and Etv5-/- embryos.

In the Control culture conditions, the ratios of PrE:EPI in Etv5-/- replicate the freshly flushed embryos in Figure 2, giving confidence there is no contamination of FGF and/or comprised development *ex vivo*.

Interestingly, we also see a failure in complete conversion of the Etv5-/- ICM to all PrE in 3 / 7 embryos. In our previous data, we had only seen this incomplete conversion in 1 / 7 embryos. Therefore, loss of Etv5 influences the effective FGF dosage cells receive. We have updated Figure 3, results and discussion section to reflect these new results.

Results

In control culture, wild-type, *Etv5*$^{+/-}$, and *Etv5*$^{-/-}$ embryos comprised of 52%, 49% and 20% GATA6+NANOG- primitive endoderm cells, respectively (Fig. 3D, E). The significant impairment of primitive endoderm specification in *Etv5*$^{-/-}$ control cultured embryos is consistent with freshly recovered *Etv5*$^{-/-}$ embryos (Fig. 2). After FGF treatment, wild-type and *Etv5*$^{+/-}$ embryos had only GATA6+NANOG- primitive endoderm cells throughout their ICM, as did half of the *Etv5*$^{-/-}$ embryos (4/7 embryos), However, some FGF treated *Etv5*$^{-/-}$ embryos retained GATA6-NANOG+ epiblast cells (3/7 embryos), restoring epiblast ratios to 41%, comparable to untreated wild-type embryos (40% epiblast). These data demonstrate that high, non-physiological levels of exogenous FGF4 can partially rescue primitive endoderm specification in *Etv5*$^{-/-}$ mutants, but cannot robustly induce a complete switch in cell-fate. Therefore, *Etv5* plays a crucial cell-autonomous role in tuning the sensitivity of ICM cells to the FGF4 signal during primitive endoderm specification.

Discussion

Exposure to high levels of exogenous FGF can partially restore primitive endoderm cell numbers in *Etv5*-deficient embryos, suggesting that related ETS family members such as *Etv1* and *Etv4* may compensate for loss of *Etv5* function under these non-physiological conditions, though a complete rescue is not achieved. Therefore, our findings support the hypothesis that *Etv5* primarily relays FGF signalling activity in uncommitted ICM cells to specify primitive endoderm.

2. In the whole paragraph starting from line 259 the authors should include the stage of the embryos they refer to as they seem to discuss both the data from E6.5 and E 6.25 embryos but this is not clear from the text.

We have updated the text to include which stages we are referring to.
Please see below for changes:

© 2025. Published by The Company of Biologists under the terms of the Creative Commons Attribution License (https://creativecommons.org/licenses/by/4.0/).

To determine if mutant embryos were specifying the AVE correctly and able to initiate gastrulation, we stained for an AVE marker, CER1, at E6.25 (pre-streak) and mesoderm marker, T (Brachyury) at E6.5 (mid-streak stage). In wild-type and *Etv4* null embryos at E6.25 and E6.5, CER1 was localised to the anterior region of the embryo, extending from the embryonic-extraembryonic junction to the distal tip (Fig. 5B, Supplemental Fig. 5A) At E6.5, T was localised to the posterior epiblast marking the nascent mesoderm (Fig. 5B). However, in single *Etv5* and double *Etv4;Etv5* homozygous mutant embryos at E6.25 and E6.5, while CER1 expression indicated that the AVE had been specified, the migration of the AVE appeared to be disrupted, delayed, or arrested at the distal tip of the embryo (Fig. 5B, Supplemental Fig. 5A, arrowheads). The occasional extension of CER1 expression beyond the embryonic-extraembryonic junction shows an over-migration of the AVE. The CER1 domain size was noticeably reduced relative to the size of the embryos. Again, abnormal thickening and disordered DVE/AVE epithelial morphology were observed in these mutants (Fig. 5B arrowhead). Finally, at E6.5, mutants were devoid of T+ cells, indicating a failure in gastrulation and primitive streak formation, and/or specification of mesoderm cell types.

3. In the paragraph starting from line 274, could the authors indicate which figure/s they refer to?

We have now indicated which figures we are referring to, updated in the text below:

The AVE migration phenotype and reduced embryo size was more severe and pronounced in compound mutant embryos $Etv4^{+/-}$;$Etv5^{-/-}$ and $Etv4^{-/-}$;$Etv5^{-/-}$ when compared with single homozygous $Etv4^{+/+}$;$Etv5^{-/-}$ embryos (Fig. 5A, B, and Supplemental Fig. 5A).

Minor points:

Fig 1C. I am assuming nr in the graph are the nr of embryos analysed. Please add explanation to the figure legend

The numbers in the graph refer to the sample size, number of analysed embryos as indicated. We have updated the figure legend accordingly.

(C) Quantification of ETV5 levels from (B). Double positive (DP; NANOG+GATA6), epiblast (EPI; NANOG+) and primitive endoderm (PrE; GATA6+). Sample size: number (n) of embryos indicated.

Fig 1 in general - I believe that supplementary fig 1C should be moved to the main fig 1. It is not clear why Fig 1 contains only pre/peri implantation stages

We have included the early post-implantation stages (E5.5 and E6.5) from supplementary Figure 1C into the main Figure 1. We also updated the original Supplementary Figure 1C channel colours of to be in line with the colours of the original Figure 1B. Thanks to the reviewers suggestion, the updated main figure now more coherently shows the expression pattern of ETV5 in embryonic and extra-embryonic endoderm lineages across early embryo development.

Supplementary Fig 2 D - colour code needs to be provided. Also, I am assuming that colour code is the same for section A-C of this figure, but this is not explicitly stated in the figure legend.

We have provided the colour code legend for Supplementary Fig. 2C and D. We also explicitly state the colours in the updated figure legend.

(A) Quantification of NANOG and GATA6 levels in an *Etv4;Etv5* allelic series of mutant embryos (from Figure 2A,B). Clustering into double positive (DP; NANOG+;GATA6+, purple), primitive endoderm (PrE; NANOG-;GATA6+, blue), epiblast (EPI; NANOG+;GATA6- light red, NANOG-;GATA6- dark red).
(B) Quantification of inner cell mass (ICM) lineage composition in an allelic series of *Etv4;Etv5* mutant embryos. Individual embryos shown and ordered by ascending cell number. Dotted line represents mean wild-type EPI:PrE composition by late blastocyst stage. (DP: purple, PrE:

© 2025. Published by The Company of Biologists under the terms of the Creative Commons Attribution License (https://creativecommons.org/licenses/by/4.0/).

blue, EPI: red)
(C) Total number of cells per embryo in DP (purple), EPI (red) and PrE (blue) lineages
(D) Total number of cells per embryo in ICM (grey) and TE (green) lineages.

Line 128; Sentence: "We collected embryos from Etv4+/-;Etv5+/- inter-cross matings at early to late blastocyst stage, and assessed the number of cells in each lineage by quantitative immunofluorescence staining for CDX2 (trophectoderm), NANOG (epiblast) and GATA6 (PrE, Supplemental Fig. 2A) as described earlier." - Supplementary fig 2A does not show TE cells

We have amended the text to state that this refers to CDX2 staining (shown in Fig. 2A) and the quantification of NANOG and GATA6 (Supplemental Fig. 2A) to assign ICM lineages.

Line 138, 141,142 - The authors refer to the wrong figure. Please correct

We have amended the text to refer to the correct figures, Fig. 2A-C.

Line 159 and 173; primitive endoerm should be corrected to primitive endoderm

We have corrected this typo throughout the text.

Line 217-8; sentence: "Analysis of published Etv5 chromatin binding in mESC (Kalkan et al., 2019) shows strong binding at a downstream enhancer region (Fig. 4D)." refers to Otx2 but it may be worth to spell it out.

We have updated the text to read:

Analysis of published ETV5 chromatin binding in mESC (Kalkan et al., 2019) shows strong binding at a downstream *Otx2* enhancer region (Fig. 4D).

It would be a good idea to include the stage of the embryo in the supplementary figure 5 B, to make it consistent with other figures.

Thank you for catching this mistaken omission, it is now amended in the updated Supplementary Figure 5.

Line 252-3; sentence: "Double knockout embryos at E6.5 were overall smaller compared to E5.5 wild-type embryos." Is the E5.5 a typo? If not, can the authors provide any data that show the comparison between double mutant E6.5 and wt E5.5 embryos?

There is a typo in the sentence, and should read "...smaller, comparable to E5.5", however we appreciate this may be confusing, so we have expanded the sentence to make the point more clearly, below:

Double knockout embryos at E6.5 were overall smaller compared to wild-type embryos at the same stage (Fig. 5), and were instead comparable in size to E5.5 wild-type embryos (Supplemental Fig. 4C).

Line 379; "After antibody staiing," it should be "staining

We have updated the typo in the text.

© 2025. Published by The Company of Biologists under the terms of the Creative Commons Attribution License (https://creativecommons.org/licenses/by/4.0/).

Second decision letter

MS ID#: dev.204278R1

MS Title: ETV4 and ETV5 Orchestrate FGF Mediated Lineage Specification and Epiblast Maturation during Early Mouse Development

Authors: Claire Simon; Woonyung Hur; Vidur Garg; Ying-Yi Kuo; Kathy K. Niakan; Anna-Katerina Hadjantonakis
Article Type: Research Article

I am happy to tell you that your manuscript has been accepted for publication in Development, pending our standard publication integrity checks.

© 2025. Published by The Company of Biologists under the terms of the Creative Commons Attribution License (https://creativecommons.org/licenses/by/4.0/).
